# Branch: an interactive, web-based tool for testing hypotheses and developing predictive models

**DOI:** 10.1093/bioinformatics/btw117

**Published:** 2016-03-07

**Authors:** Karthik Gangavarapu, Vyshakh Babji, Tobias Meißner, Andrew I. Su, Benjamin M. Good

**Affiliations:** ^1^Department of Molecular and Experimental Medicine, The Scripps Research Institute, La Jolla, CA 92037, USA; ^2^Avera Health, Sioux Falls, South Dakota, USA

## Abstract

**Summary:** Branch is a web application that provides users with the ability to interact directly with large biomedical datasets. The interaction is mediated through a collaborative graphical user interface for building and evaluating decision trees. These trees can be used to compose and test sophisticated hypotheses and to develop predictive models. Decision trees are built and evaluated based on a library of imported datasets and can be stored in a collective area for sharing and re-use.

**Availability and implementation:** Branch is hosted at http://biobranch.org/ and the open source code is available at http://bitbucket.org/sulab/biobranch/.

**Contacts:** asu@scripps.edu or bgood@scripps.edu

**Supplementary information:** Supplementary data are available at *Bioinformatics* online.

## 1 Introduction

Scientific advances based on large datasets often involve a cycle of interactions between experts in computation (‘data scientists’) and experts in the domain of inquiry. Typically, computational scientists will need to adapt scripts for data analysis and presentation at each step, limiting the number of potential iterations and resulting in relatively little feedback from the subject matter expert. Graphical interfaces for data exploration can empower domain experts with the ability to engage with data directly, increasing their capacity to rapidly define and answer questions leading to new insights (Hinterberg *et al.*, 2015).

**Fig. 1.**
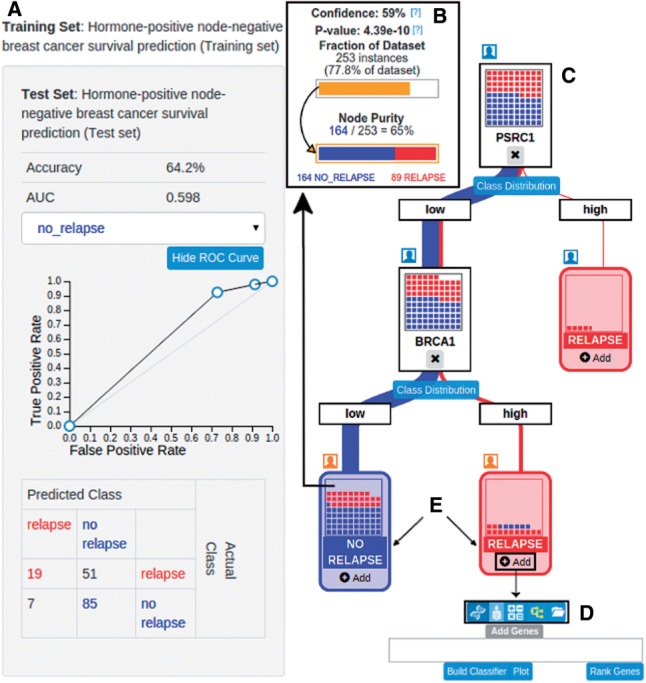
A decision tree built using a node-negative, ER-positive, HER2-negative breast cancer dataset (Griffith *et al.*, 2013). (**A**) Evaluation of the tree on the testing set. The evaluation sidebar shows the accuracy, area under the curve and the confusion matrix. (**B**) Pop-up window characterizing the ‘Low PSRC1 and Low BRCA1’ leaf node in terms of the fraction of the dataset that reaches the node, its relative class purity and the chances of obtaining a split of the dataset with that purity by chance. (**C**) The full decision tree as visualized in Branch. (**D**) The search bar used to add split nodes to the decision tree. (**E**) ‘Relapse’ and ‘No Relapse’ are the two class labels in the specified training and test sets

Given a dataset with many features (e.g. gene expression measurements, clinical attributes) and nominal class values for each sample (e.g. breast cancer relapse status), a decision tree can provide a visual representation of a sophisticated logic function linking selected input features to an output class. Decision trees are often induced automatically from training data and applied to classify new samples. They can also be constructed manually, with the intent to either incorporate domain expertise into their structure to improve generalizability (Stumpf *et al.*, 2009) or to test specific hypotheses. As one example of the latter process, a researcher may ask whether a dataset supports her hypothesis that breast cancer relapse will be associated with high levels of *PSRC1* expression or a combination of low levels of *PSCR1* and high levels of *BRCA1*. This hypothesis can be represented as a decision tree rooted in a feature for *PSRC1* gene expression with a leaf node predicting relapse for high *PSCR1* and a secondary split under low *PSCR1* on *BRCA1* expression (Fig. 1). The extent to which this decision tree fits the dataset, measured by e.g. its predictive accuracy or a statistical test of association between samples in leaf nodes and their actual class values (Fig. 1B), indicates the extent to which the data supports the hypothesis. Here we introduce an interactive, collaborative Web application, called Branch, that allows domain experts to rapidly and easily build, evaluate and share decision trees based on large biomedical datasets.

## 2 Using Branch

### 2.1 Dataset library

Users begin by selecting a dataset from the Branch dataset library. Each dataset corresponds to a single table where each row contains the values for a set of features, e.g. gene expression levels or clinical variables, and a binary class label for each sample, e.g. cancer/normal. The Branch library currently contains several datasets selected to demonstrate the features of the application. Additional datasets may be loaded upon request. Branch may also be installed locally from its open source code, allowing the user to load their own datasets.

### 2.2 Evaluation

Once a dataset is selected, the user must choose a process for evaluating the trees that they will build. In using the tool to iteratively create, test and adapt decision trees, the user can act much like a learning algorithm. As in machine learning, the choice of evaluation process can be used to reduce the likelihood of overfitting the data. The user may select from three options: ‘test set’, ‘percentage split’ and ‘training’ (Supplementary Fig. S1). When distinct training and testing sets are available the ‘test set’ option enables the user to build their trees using feedback, such as feature rankings, from the training set while seeing the global evaluation metrics (AUC, Accuracy, etc.) based on the tree’s performance on the unseen test set. When no independent test set is available, this process may be simulated using the ‘percentage split’ option. Finally, users that are only interested in seeing precisely how a tree fits a dataset may choose the ‘training set’ evaluation function.

### 2.3 Building trees

Given a dataset and an evaluation method, the user can begin constructing decision trees and measuring their quality (Fig. 1). Building a tree corresponds to the process of iteratively adding split nodes. Branch supports five different split node types. Most simply, single feature splits such as the split on *PSRC1* in Figure 1 may be created from individual features such as the expression values for a particular gene or the age of a patient (Supplementary Fig. S2). Custom features may be created as linear combinations of other features. For example, the OncotypeDx (Paik *et al.*, 2004) breast cancer recurrence score can be recreated and applied as a feature for use in single Branch split nodes (Supplementary Section S3, Supplementary Fig. S3–S6). The user may also choose to use built-in machine learning algorithms to infer a predictive model from a feature subset and use the model for a decision node (Supplementary Fig. S7). Likewise, previously created trees can be used as individual decision nodes. Finally, the system provides a visual split creator that lets the user define decision boundaries graphically (Supplementary Fig. S8) (Ware *et al.*, 2001). A tree may incorporate mixtures of these different node types.

Users can begin building a tree from scratch or can select an existing tree from the community library or their personal collection. Once created, the user may save their tree to the public collection or keep it private. Branch is specifically useful for the analysis of large biological datasets with well-defined class values. In cases where users may want to define their own complex classes, a related tool for user-guided decision tree analysis called Peax may be more appropriate (Hinterberg *et al.*, 2015).

## 3 Conclusion

Branch provides a new mechanism to connect a large pool of biologically savvy (but perhaps not computationally savvy) researchers with large, high-dimensional datasets. The rich, graphical user interface reduces barriers between researchers and data, stimulating rapid, direct exploration of previously opaque yet valuable information. In addition, Branch is uniquely collaborative, providing an unprecedented avenue for researchers to share their hypotheses and their predictive models with the community and to build on the work of others.

### 3.1 Implementation

Branch is available online at http://biobranch.org with open source code available at http://bitbucket.org/sulab/biobranch. It consists of a Java Spring server application that takes advantage of the Weka (Frank *et al.*, 2004) machine learning library and a Web client application based on Backbone.js and d3.js (Bostock *et al.*, 2011).

## Supplementary Material

Supplementary Data
