# Supplementary material for: Branch: an interactive, web-based tool for testing hypotheses and developing predictive models
Source: Bioinformatics. 2016 Mar 7;32(13):2072–4. doi: 10.1093/bioinformatics/btw117 (PMC4920125; doi:10.1093/bioinformatics/btw117)
Supplement: Supplementary Data [file supp_32_13_2072__index.html]

Branch: an interactive, web-based tool for testing hypotheses and developing predictive models — Supplementary Data 

# Branch: an interactive, web-based tool for testing hypotheses and developing predictive models

## Supplementary Data

files

- Supplementary Data - pdf file
