# Supplementary material for: Branch: an interactive, web-based tool for testing hypotheses and developing predictive models
Source: Bioinformatics. 2016 Mar 7;32(13):2072–4. doi: 10.1093/bioinformatics/btw117 (PMC4920125; doi:10.1093/bioinformatics/btw117)
Supplement: Supplementary Data [file supp_btw117_Supplementary_resubmission.pdf]

Supplementary Material for

Branch: An interactive, web-based tool for testing hypotheses and developing predictive models

Karthik Gangavarapu
Vyshakh Babji
Tobias Meißner
Andrew I. Su
Benjamin M. Good

Branch (http://biobranch.org) is a collaborative Web application that enables users to manually construct and evaluate sophisticated decision trees with large biomedical datasets. The following supplementary data provides additional information in support of the manuscript "**Branch: An interactive, web-based tool for testing hypotheses and developing predictive models**".

Table of contents

1. Evaluation options
2. Creating a split node
3. Creating the OncotypeDx (Paik et al., 2004) breast cancer recurrence score as a Custom Feature
4. Building a Classifier Split Node and using the 'Plot' interface
5. Using Branch to evaluate hypotheses
6. References

1 Evaluation Options

There are three different evaluation options available in Branch. Fig 1 shows the three options along with a brief summary as shown to the user in the online interface.

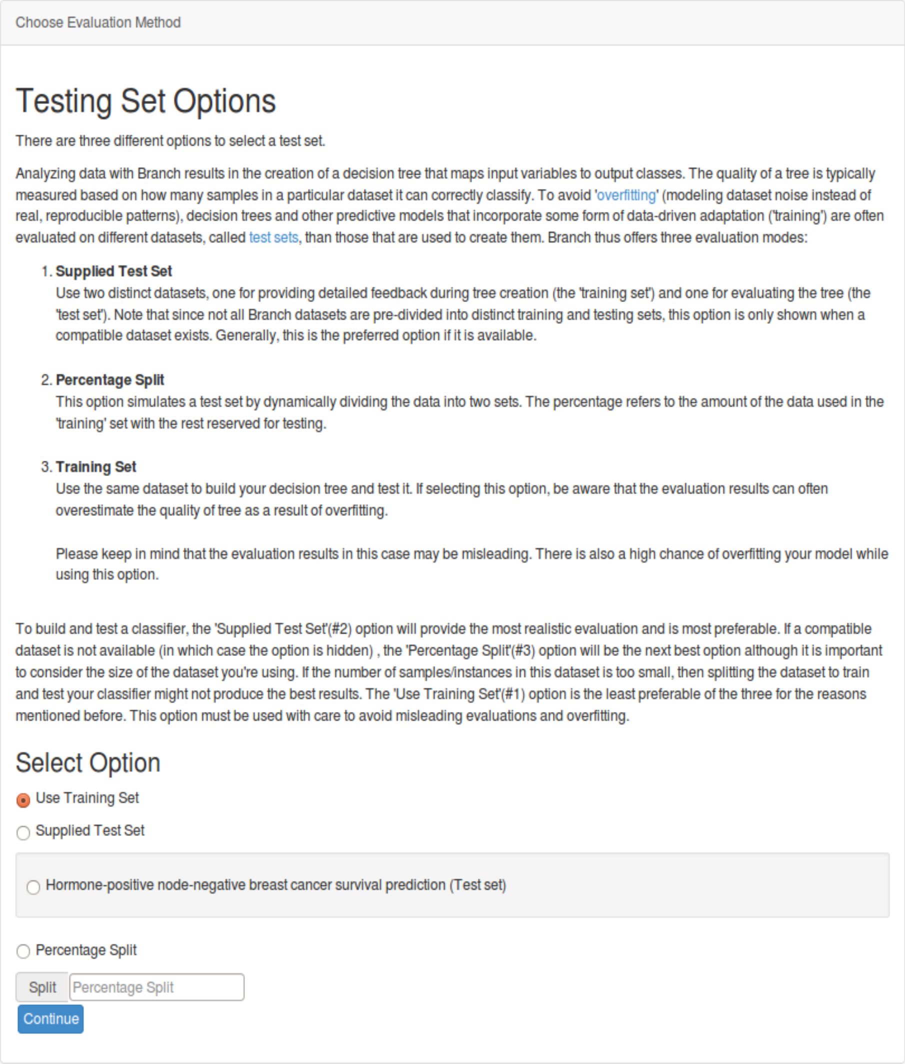

Figure 1: Evaluation Options. Branch presents the user with three options for evaluating decision trees: test set, percentage split, and training set.

2 Creating a split node

The steps involved in creating a split node are described in Fig 2. The online interface presents documentation and video demonstrations of adding the various kinds of split nodes. These can be accessed via a the Help link at the top right of the application.

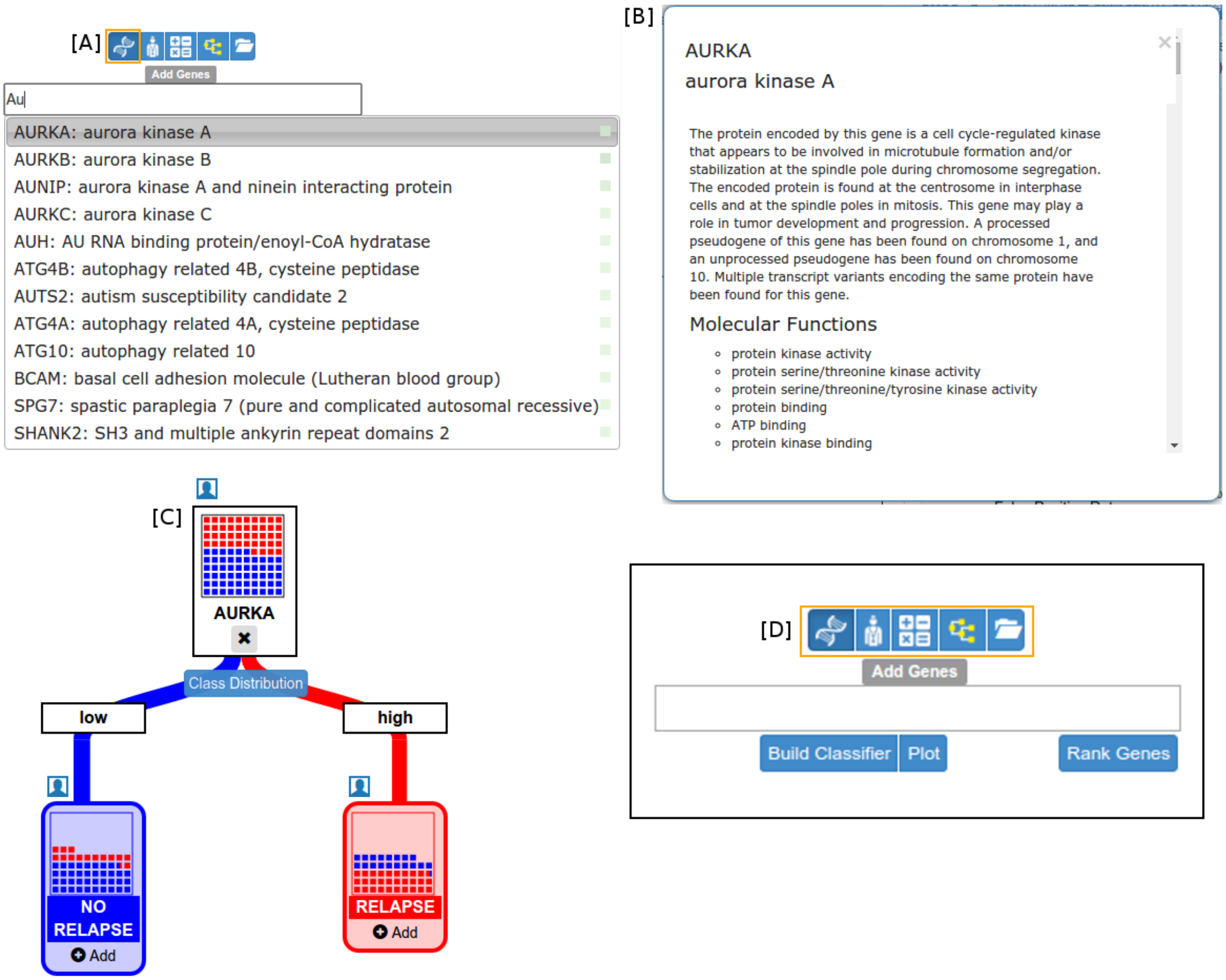

Figure 2: Steps to add a split node. [A] Choose the first icon on top of the search bar to select genes. Begin typing the gene name in the autocomplete box and pick the required gene from the drop down. Notice the green colored squares to the right of every option. This color is representative of the predictive capacity of each option with respect to the instances (from the training set) in the node. Greater the predictive power, darker the green. [B] Hovering over the options in the dropdown produces a popup with further information regarding the option. For example, when searching for genes, the user is presented with a gene summary followed by Gene Ontology annotations and GeneRIFs. [C] Choosing an option from the autocomplete dropdown will produce a split node as shown. [D] Each of the five icons on top of the search bar represent a kind of split node that can be added. From left to right, they represent, genes, non-genes (e.g. clinical features), custom features, classifiers and previously built trees on Branch.

3 Creating the OncotypeDx breast cancer recurrence score as a Custom Feature

The Oncotype Dx test uses a linear combination of gene expression values to predict the likelihood of breast cancer relapse (Paik *et al*., 2004). The recurrence prediction algorithm uses the expression values of 21 genes divided into groups as shown in Figure 1. The reference group is a set of five genes used to

normalize the group scores. Below, we show how to precisely reproduce the recurrence score using the “custom feature” option available in the Branch split builder.

Proliferation Group
Ki67
STK15
Survivin
CCNB1
MYBL2

Invasion Group
MMP11
CTSL2

GSTM1

BAG1

CD68

HER2 Group
GRB7
HER2

Estrogen Group
ER
PGR
BCL2
SCUBE2

Reference Group
ACTB
GAPDH
RPLPO
GUS
TFRC

Figure 3: **Panel of 21 genes.**

Individual Group Scores

1. Ref Group Score = 16 - ((ACTB + GAPDH + RPLP0 + GUS + TFRC)/5)
2. HER2 group score = 0.9 x GRB7+ 0.1 x HER2 (if the result is less than 8, then the GRB7 group score is considered 8)
3. ER group score = ((0.8x ER +1.2 x PGR + BCL2+ SCUBE2)/4)
4. Proliferation group score = ((Survivin + KI67 + MYBL2 + CCNB1 [the gene encoding cyclin B1]+ STK15)/5) (if the result is less than 6.5, then the proliferation group score is considered 6.5)
5. Invasion group score=((CTSL2 + MMP11)/2)
6. GSTM1 group score = GSTM1
7. BAG1 group score = BAG1
8. CD68 group score = CD68

Recurrence-Score Algorithm

Recurrence Score(RS) = 0.47 * HER2 group score - 0.34 * ER group score + 1.04 * PROLIFERATION group score + 0.10 * INVASION group score + 0.05 * CD68 - 0.08 * GSTM1 - 0.07* BAG1

Generating a Recurrence-Score Feature in Branch

To re-create the recurrence-score algorithm using Branch, we first build each of the group scores (using the set of five reference genes for normalization) as individual "custom features". These individual custom features are then combined together using the recurrence-score algorithm, with the relevant limits and weights in place, to build the recurrence score (Figure 2). For certain group scores, namely the ER group and the Proliferation group, if the group score is less than a certain limit, the limit itself is considered to be the group score. This limit can be specified using the slider/textbox (Figure 3A). Once saved, the new feature can be picked using the autocomplete search bar and added as a split node to the tree (Figure 3C). Please note that the "Custom Features" category must be chosen using the icons above the search bar to select custom features.

We validated that the recurrence score feature created within Branch exactly reproduces the recurrence score utilized in the R code provided in the supplementary data from (Griffith *et al,*. 2013). When working with a breast cancer dataset in the current deployment, the user can use the recurrence score feature to construct their decision trees. A decision tree using this custom feature can be found at http://biobranch.org/?treeid=404260&dataset=288557 and one with the pre computed recurrence score using R code (picked as a non-gene feature) can be found at http://biobranch.org/?treeid=404266&dataset=288557 (both trees can be accessed by logging into Branch anonymously or with an account).

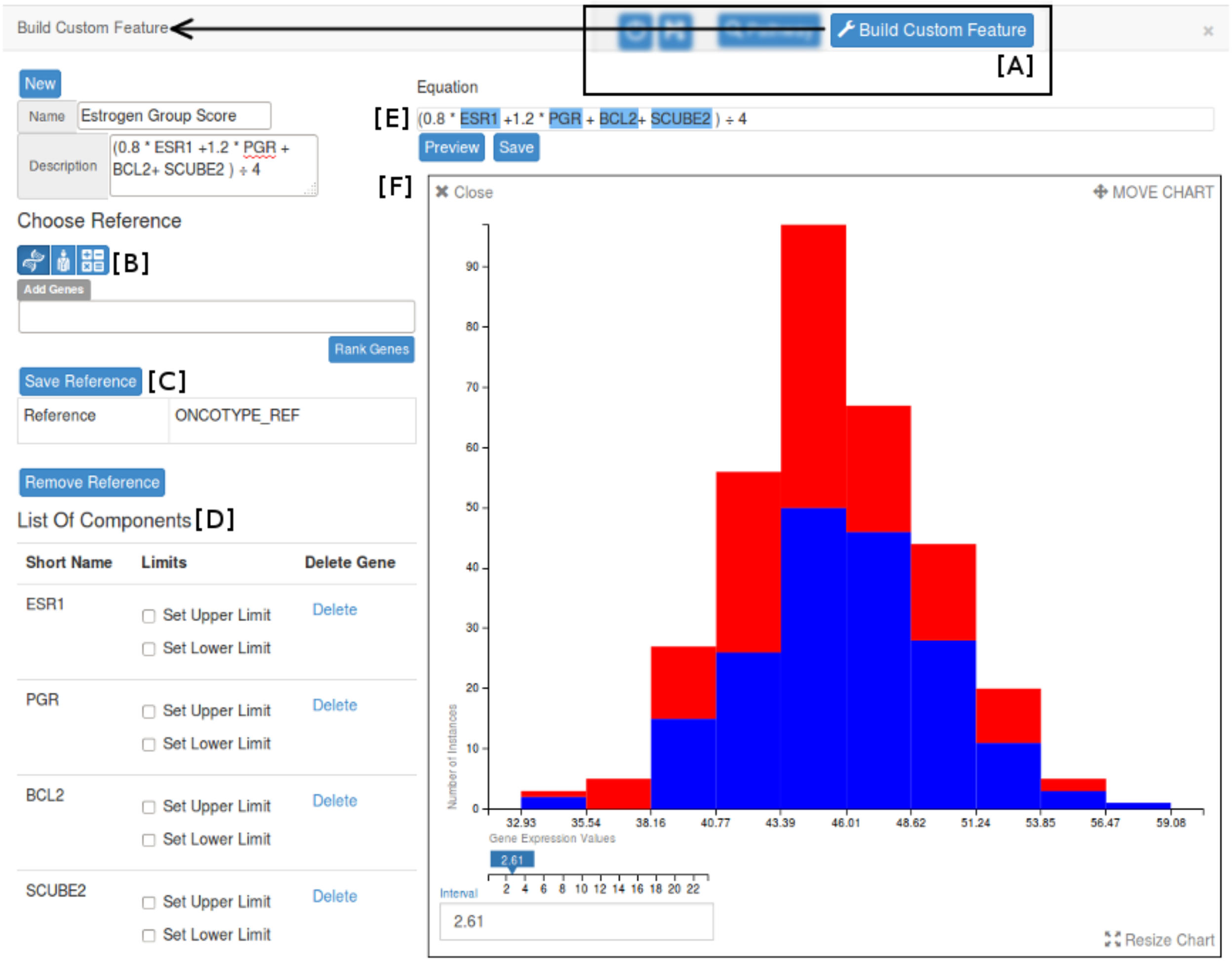

Figure 4: **Building a custom feature in Branch.** [A] Click on the "Build Custom Feature" button on the right sidebar to open the window. [B] Use the autocomplete search bar to pick dataset attributes to be used as components of the custom feature. [C] Since the "Ref group score" is added to each of the genes in the group scores, you can create a custom feature using the equation for Ref group score and simply add it as a "Reference" for the subsequent custom features and the value is automatically added without having to repeat the same equation every time. [D] This list shows attributes that have been chosen to be added as "components" to the custom feature. [E] The mathematical equation combining the chosen components can be typed here. As the equation is typed out, the components are highlighted to indicate that the required attributes in the dataset have been identified. [F] Using the "Preview" button will show the distribution of the new custom feature that has been built. If the equation is invalid then an error message is displayed.

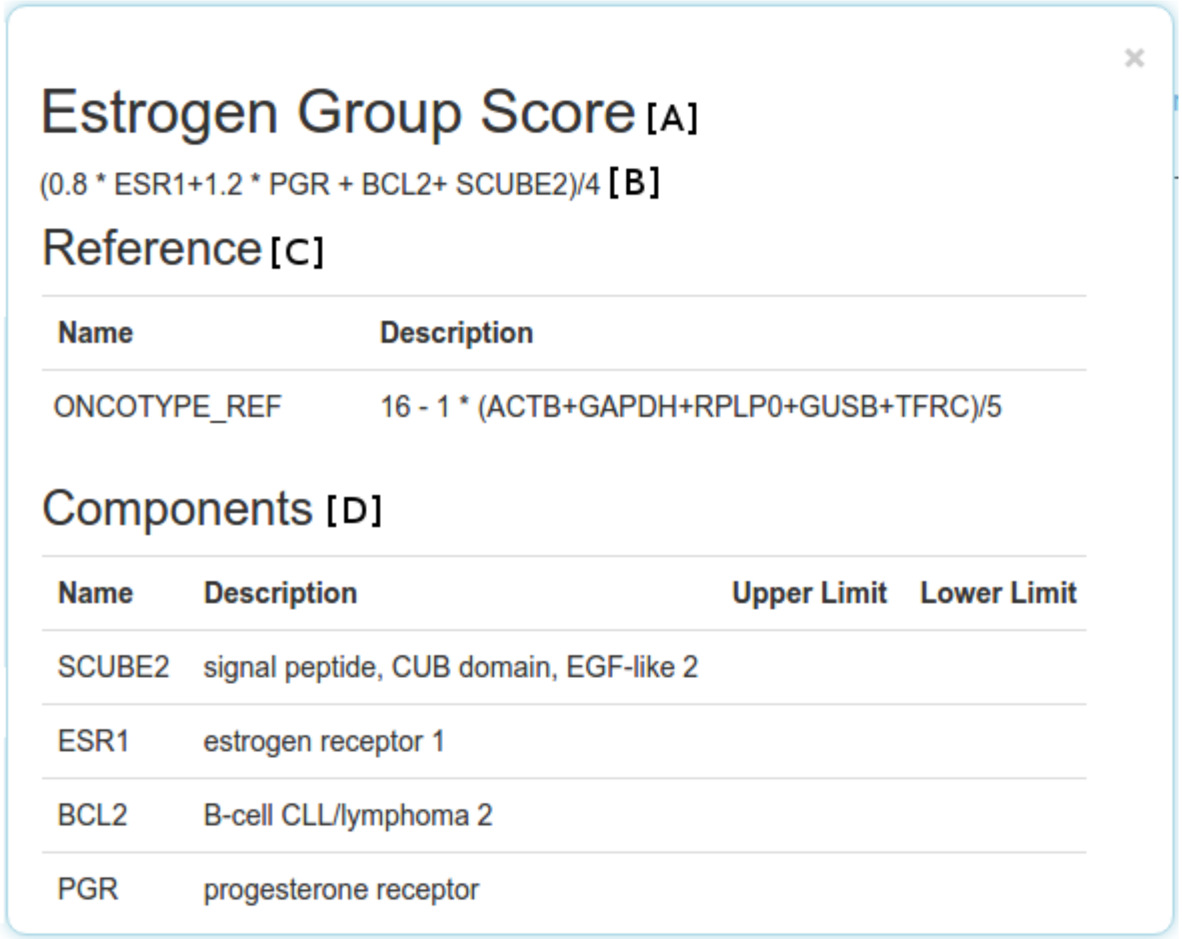

Figure 5: Details of a Custom Feature. These details can be viewed in Branch by adding the custom feature to a tree and clicking on the split node title. [A] Name of the custom feature. [B] Description of the custom feature (equation used to generate it). [C] The attribute (may be a gene, non-gene or a custom feature) used as a reference in the custom feature. The reference is automatically added to the equation (Fig 2E) of the custom feature to give the final result. [D] List of components used in the equation (Fig 2E) of the custom feature.

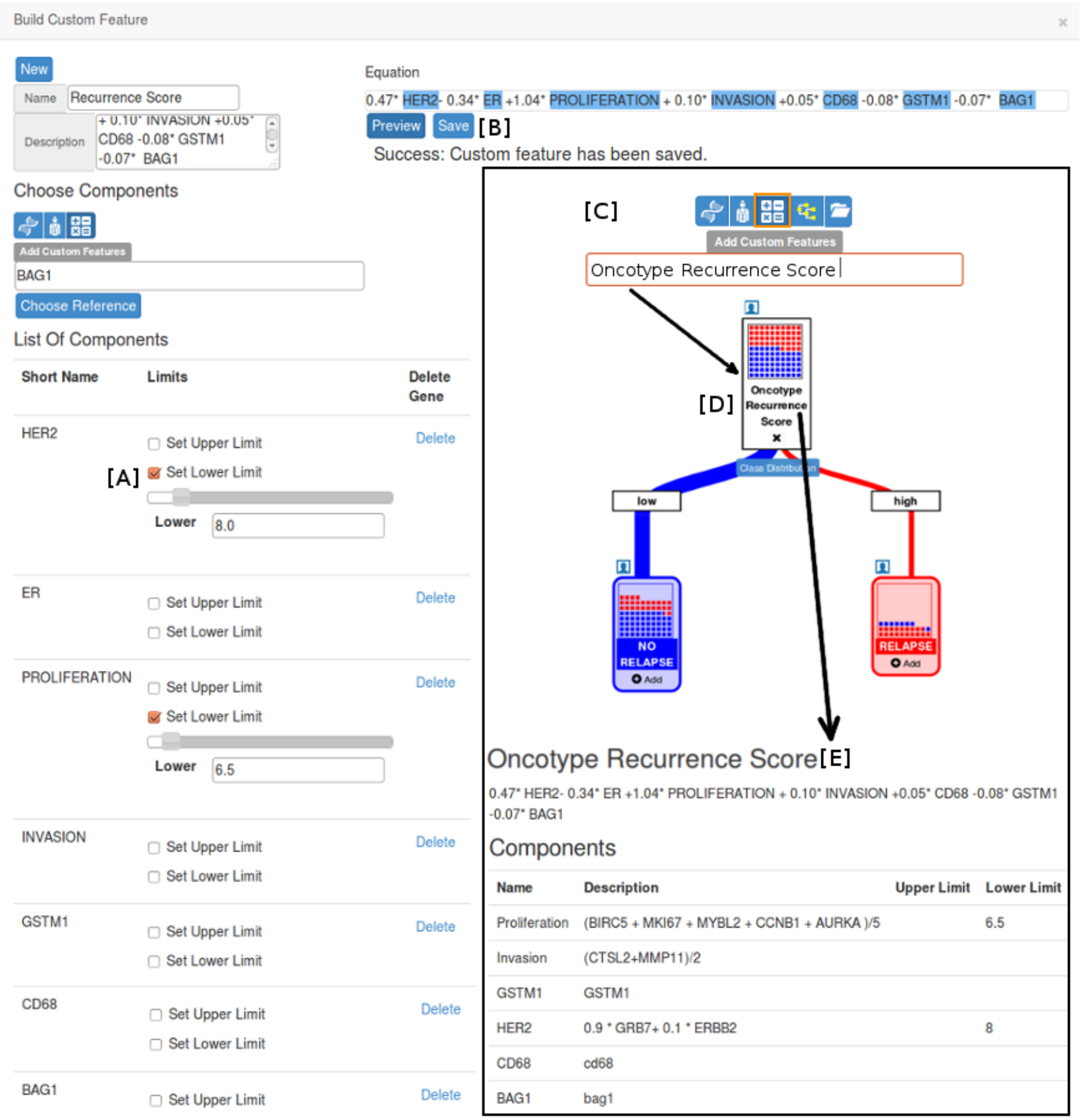

Figure 6: Saving and using a custom feature. [A] Specifying the bounds on a custom feature. [B] After the "custom feature" has been built and previewed, it must be saved using the "Save" button. [C] The "custom feature" category can be selected by clicking on the highlighted icon. The autocomplete tool can then be used search for and pick the newly created custom feature and add it to the tree. [D] Once chosen, the split node is added to the tree. [E] Clicking on the title in the split node will show the description of the custom feature, the attributes used as components of the custom feature and the limits specified, if any.

4 Building a Classifier Split Node and using the 'Plot' interface

Fig 7 outlines the steps involved in choosing a set of features to train a classifier and using that classifier to build a split node in the tree.

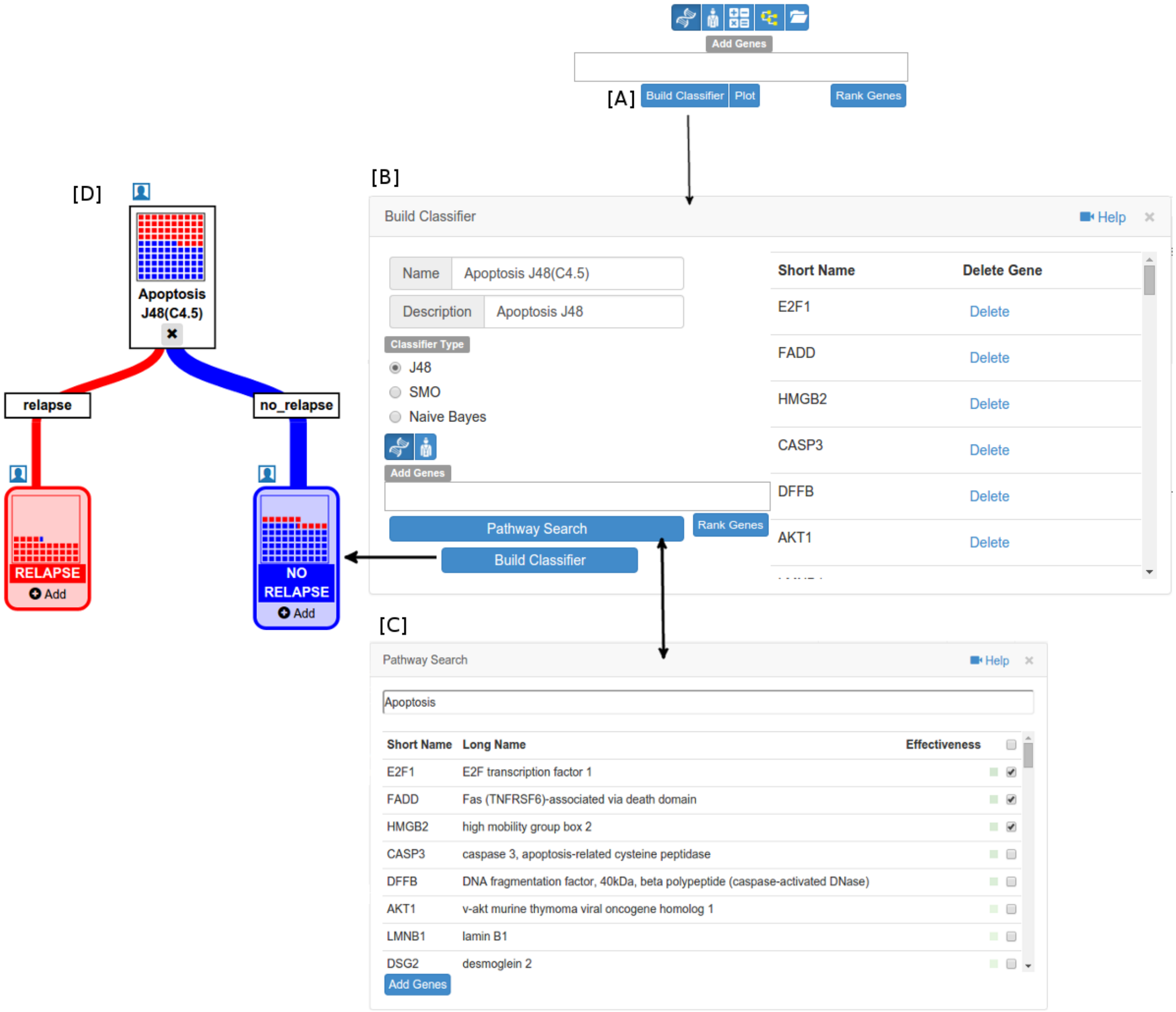

Figure 7: Building a 'classifier' split node. [A] The 'Build Classifier' option can be used to build a classifier using instances at any node. [B] In this window, the user can specify a name, description and select a type of classifier followed by attributes required to build the classifier. Each of the classifier types use Weka library implementations. J48 is an implementation of the C4.5 decision tree induction algorithm and SMO is a support vector machine implemented by 'Sequential Minimal Optimization'. The input features for the classifier can be selected using the autocomplete interface or the 'Pathway Search'. [C] Pathway search can be used to find genes related to specific biological pathways. Genes can be selected by using the checkbox to the right of each gene. To select/unselect all genes, the checkbox in the header can be used. The pathway and related genes are pulled from ConsensusPathDb (Kamburov, A. *et al.*, 2013). [D] The split node produced using the classifier that was trained using instances in the node.

Branch can be used to plot instances using two attributes from the dataset. The user can use these plots to visually select specific instance groups to build a split node. Fig 8 outlines the steps involved in building such a split node.

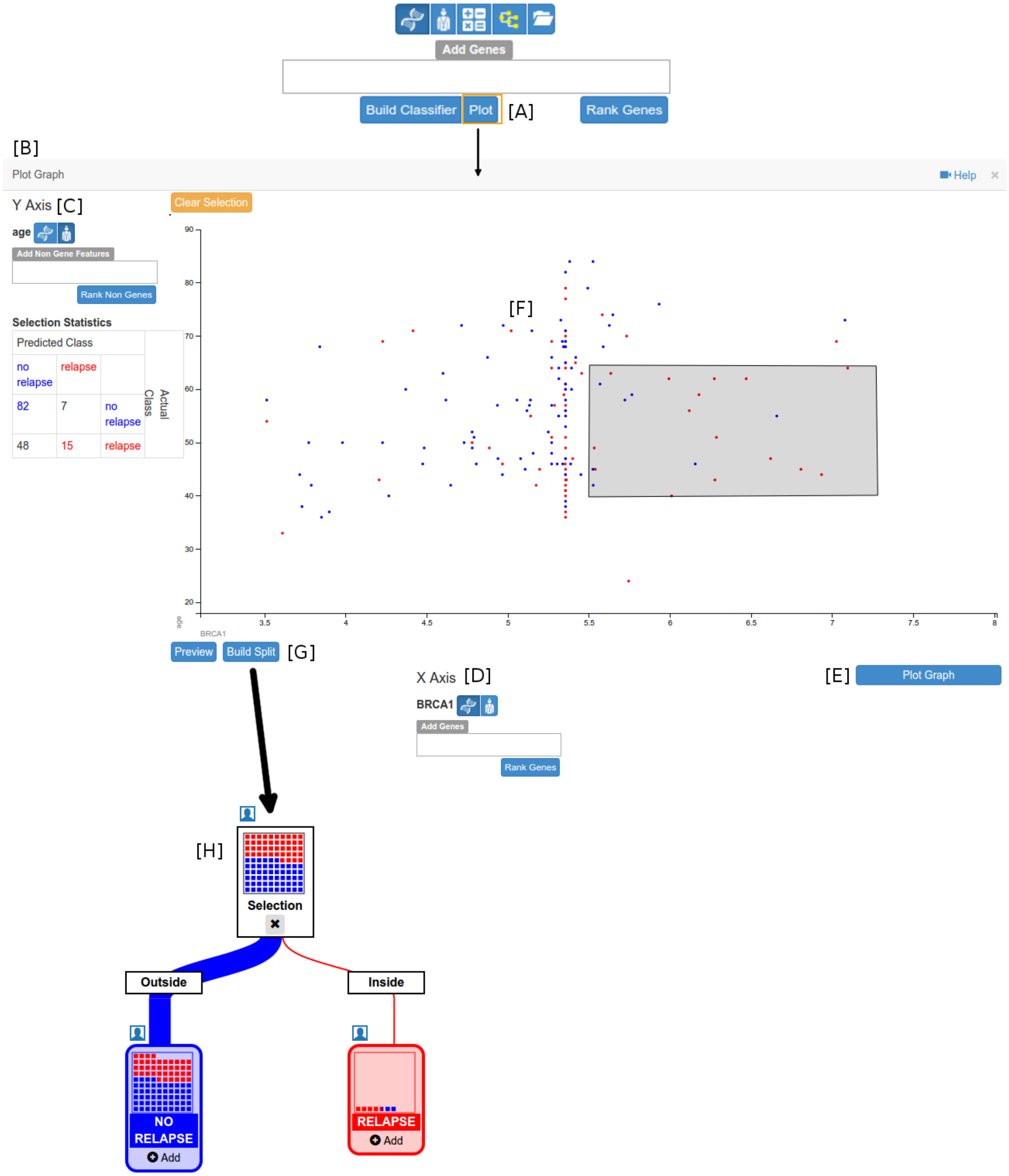

Figure 8: Selection of samples between ages of 40 and 65 and high BRCA1 expression by using 'Plot'. [A] 'Plot' can be used to select a decision boundary within 2-feature view of the training data. [B] Clicking on 'Plot' produces the plot interface. [C] The feature to be used as the Y axis can be selected

using the standard autocomplete interface. [D] Attribute to be used as the X axis can be selected using the same autocomplete interface. [E] Once the X and Y axes have been selected, 'Plot Graph' can be used to render the instances in a chart. [F] Instances can be selected using a polygon selector. [G] Clicking 'Build Split' will add the selection as a split node to the tree. [H] The split node produced from the selection. All selected instances will appear in the 'Inside' branch and all the other instances in the 'Outside' branch.

5 Using Branch to answer questions and evaluate hypotheses

This section shows how Branch can be used to interrogate a breast cancer dataset to answer specific questions related to biological hypotheses. The training set used is "Hormone-positive node-negative breast cancer survival prediction (Training set)" and the test set is "Hormone-positive node-negative breast cancer survival prediction (Test set)". Both datasets are available to use on the online interface.

Question 1: **If AURKA expression is high and TOP2A expression is low, are the chances of relapse lower or higher?** The decision tree depicted in Figure 9 and accessible online at http://biobranch.org/?treeid=405257&dataset=288557 models this gene expression pattern in the context of samples from breast cancer patients that either had a relapse before 10 years (high risk) or did not (low risk). This tree would predict that high AURKA and low TOP2A would be in the low-risk, no-relapse group; however, the quality of that leaf node is so low (54% accurate) that it provides effectively no support for the hypothesis. A Fisher's exact test conducted on a contingency table based on the samples that follow the pattern in the rule (are a member of the set in the leaf node) versus the rest of the samples (not in the leaf node) as compared to the correct classifications of all of the samples produces a P value of 1, confirming the lack of support for the hypothesis (**Table 1, Fig 9D**).

Table 1: Contingency table for the rule If AURKA expression is high and TOP2A expression is low, then predict No Relapse in the context of the (Griffith et al 2013) training dataset. The P value according to Fishers exact test = 1, indicating that the rule should be rejected in favor of the null hypothesis.

	No relapse	Relapse	Totals
In node: (AURKA high, TOP2A low)	7	6	13
Out of node	175	137	312
Totals	182	143	325

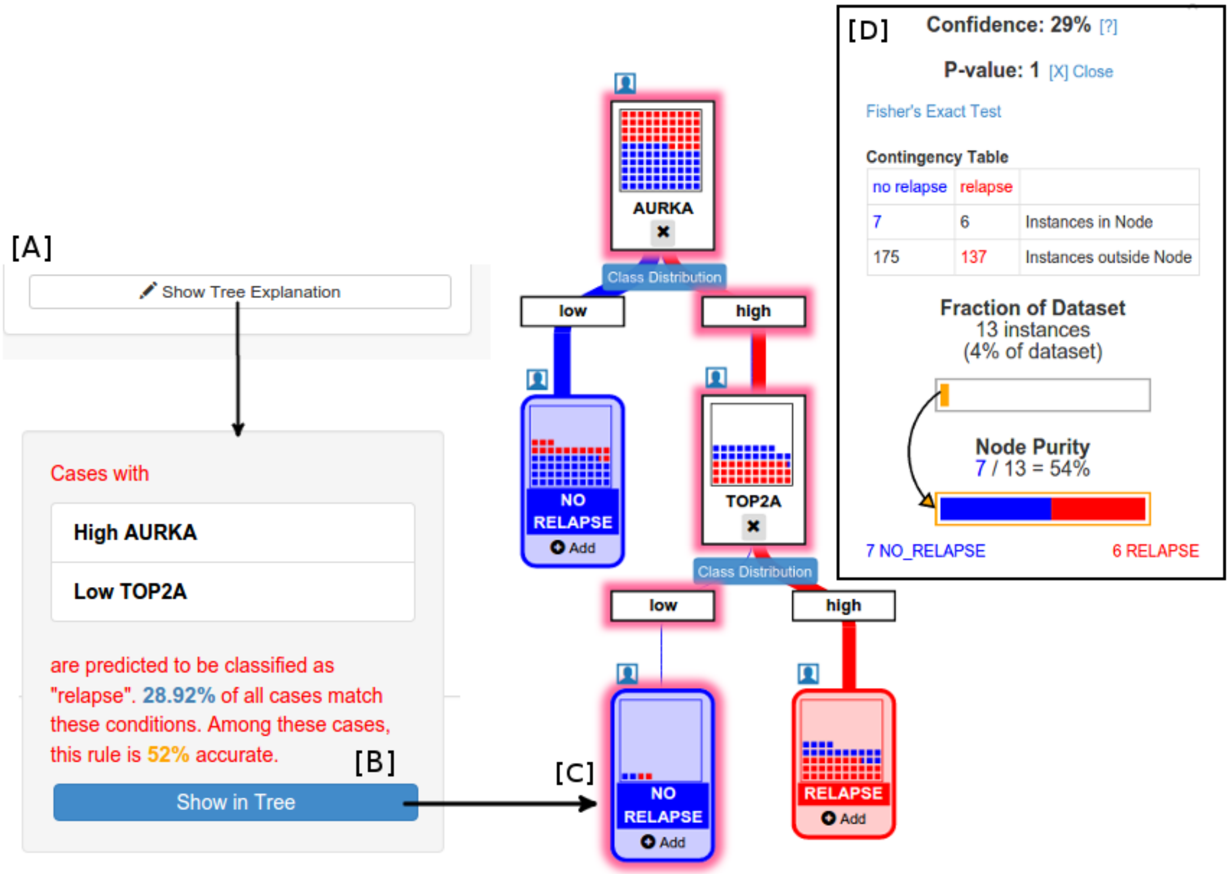

Figure 9: If AURKA expression is high and TOP2A expression is low, is the risk of recurrence (relapse/no relapse) lower or higher? [A] After building a tree, clicking on 'Show Tree Explanation' will show a more descriptive explanation of each node in the tree. [B] Clicking on the 'Show in Tree' button will highlight the corresponding node and the flow of instances from the root node with a 'pink' border. [C] The corresponding node contains instances with high expression of AURKA and low expression of TOP2A. [D] Clicking on the "No Relapse" node shows the confidence, P value, the contingency table used for the Fisher's Exact Test to calculate P value, the fraction of dataset in the node and the node purity.

Question 2: **Is the OncoTypeDx recurrence score predictive of recurrence?** Following the same pattern as above, a decision tree is constructed that is rooted in the OncoTypeDx feature (Figure 10). Inspection of the leaf nodes confirms that, in this dataset, the feature is predictive (Table 2).

Table 2: Contingency table for the rule If OncotypeDx Recurrence score is high, then predict Relapse in the context of the (Griffith et al 2013) training dataset. The P value according to Fishers exact test is 6.296E-9, suggesting that the rule is significant and the null hypothesis should be rejected(**Fig 9**).

	Relapse	No relapse	Totals
In node: (RS high)	61	25	86
Out of node: (RS low)	82	157	239
Totals	143	182	325

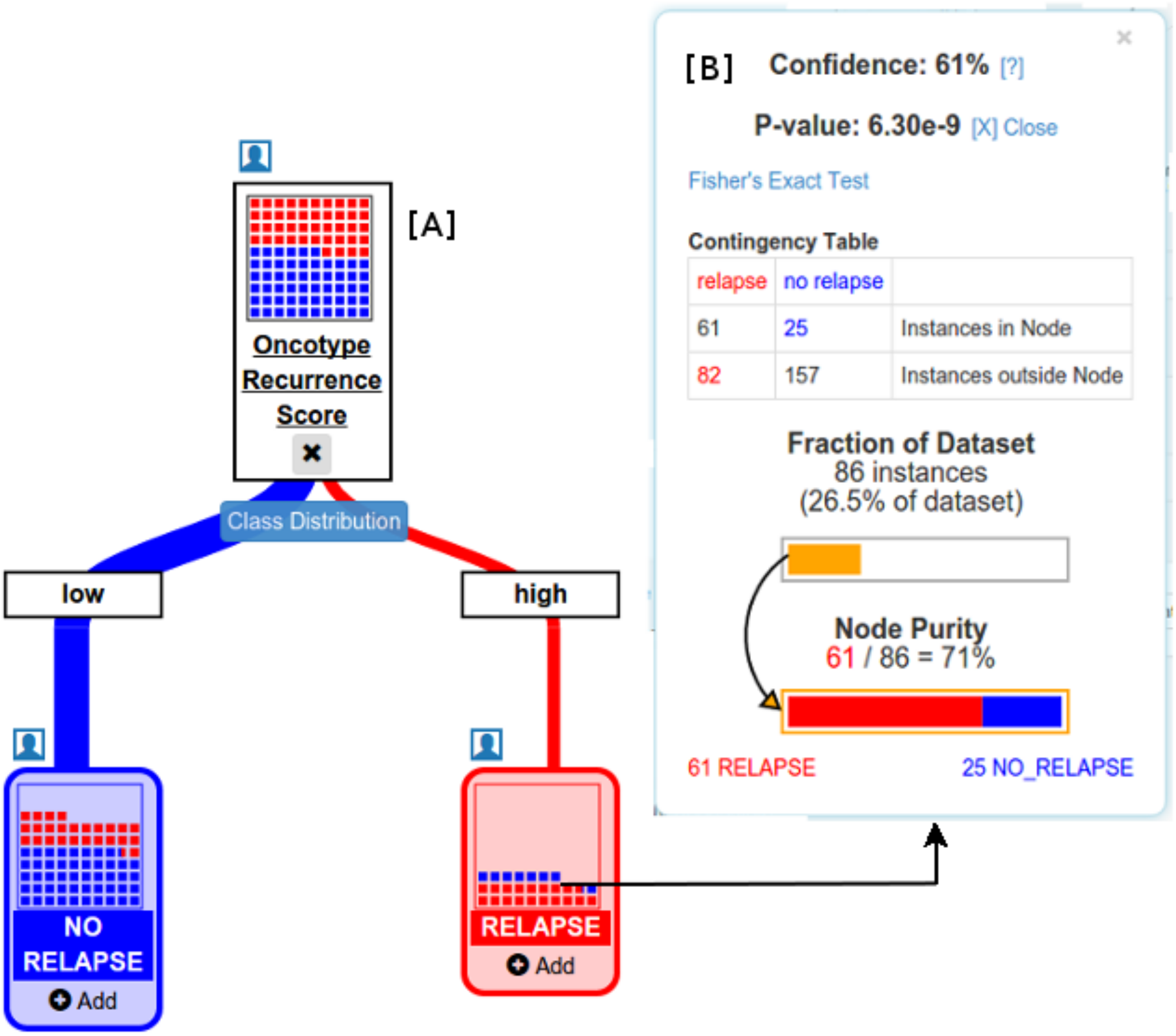

Figure 10: Oncotype Recurrence Score on Branch. [A] Oncotype Recurrence Score 'split node' constructed as shown in Fig 3-6. [B] Clicking on the "Relapse" node shows the P value and the contingency table used for the Fisher's Exact Test.

Is the expression of VEGFA associated with breast cancer relapse?

Figure 11 follows the same pattern as above, showing that, in the context of this dataset there appears to be no direct relationship between VEGFA and relapse.

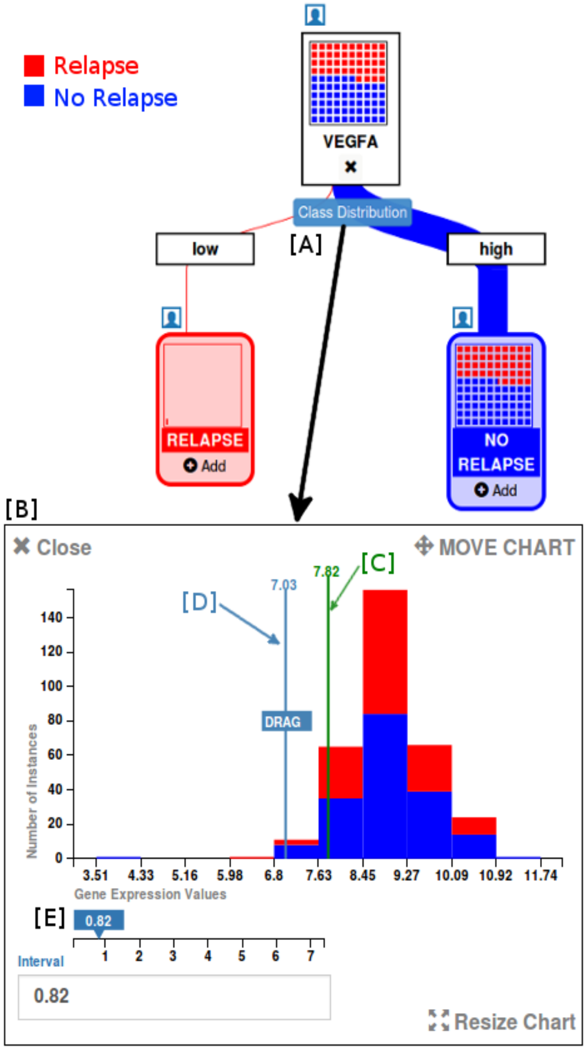

Figure 11: Do the expression levels of VEGFA correlate with survival? [A] After adding the split node, clicking on 'Class Distribution' will show the distribution of attribute values and class values. [B] The chart shows the distribution of the attribute values (VEGFA Gene Expression Values) in specific bins. [C] The 'split value' picked by the application to produce 'low' and 'high' nodes. [D] By dragging this bar the user can shift the distribution of instances in the 'low' and 'high' nodes as required. [E] The bin size (Gene Expression Values) in the graph can be modified by dragging the bar.

Aside from fairly simple assessments based on individual features, the more advanced Branch split node types offer the potential to rapidly explore more complex questions. For example, consider the question **"Is the expression of genes in the Caspase Cascade pathway more or less predictive of relapse than those involved in Mitochondrial Biosynthesis pathway?"** The 'classifier split node' (Fig. 7) can be used to rapidly assemble a split based on all the genes in either pathway (Figure 12, 13). It can be seen that while using the specified training and test sets, genes from the Caspase Cascade Pathway are more predictive than those in the Mitochondrial Biosynthesis Pathway.

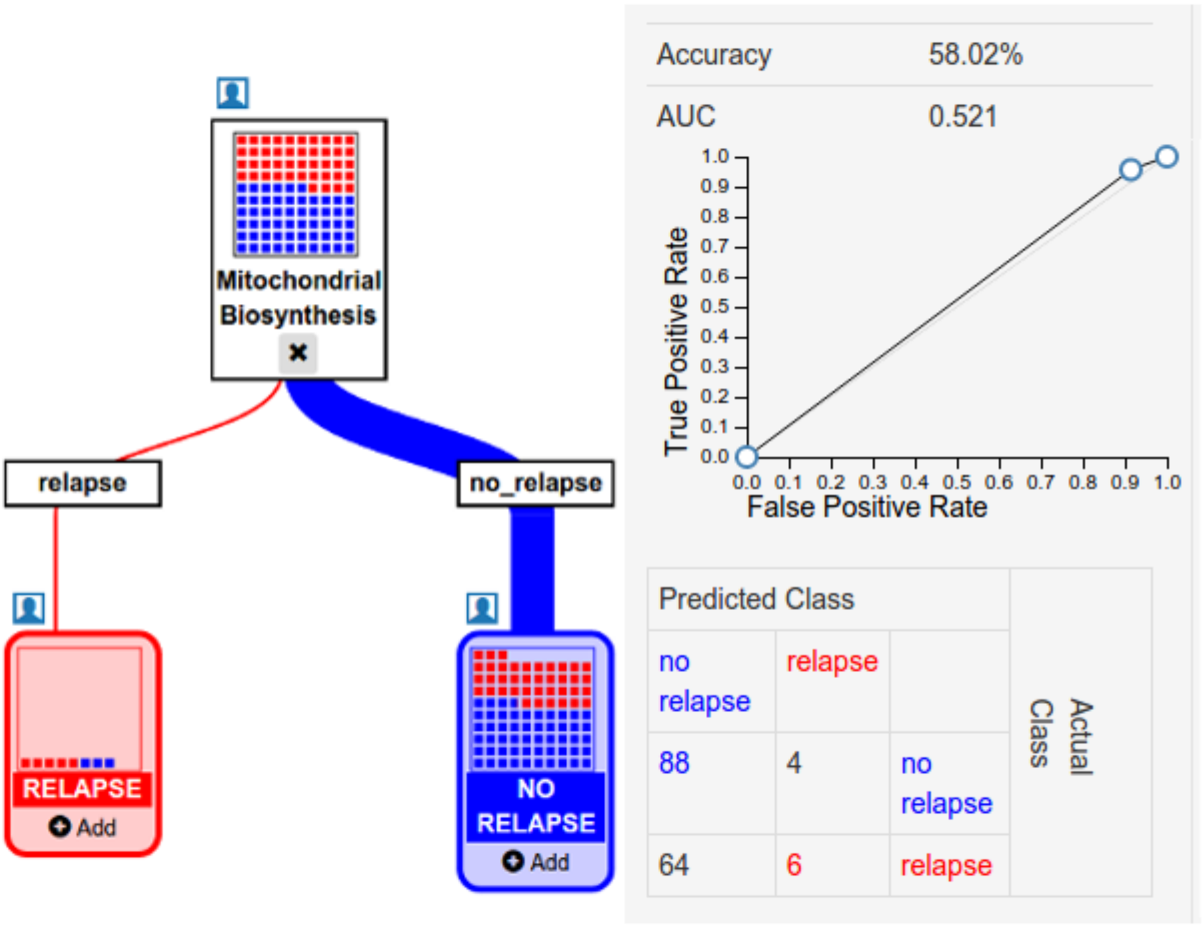

Figure 12: A SVM Classifier Split Node constructed from the Mitochondrial Biosynthesis Pathway (link). Training Set: "Hormone-positive node-negative breast cancer survival prediction (Training set)". Test Set: "Hormone-positive node-negative breast cancer survival prediction (Test set)". The classifier has an accuracy of 58.02%.

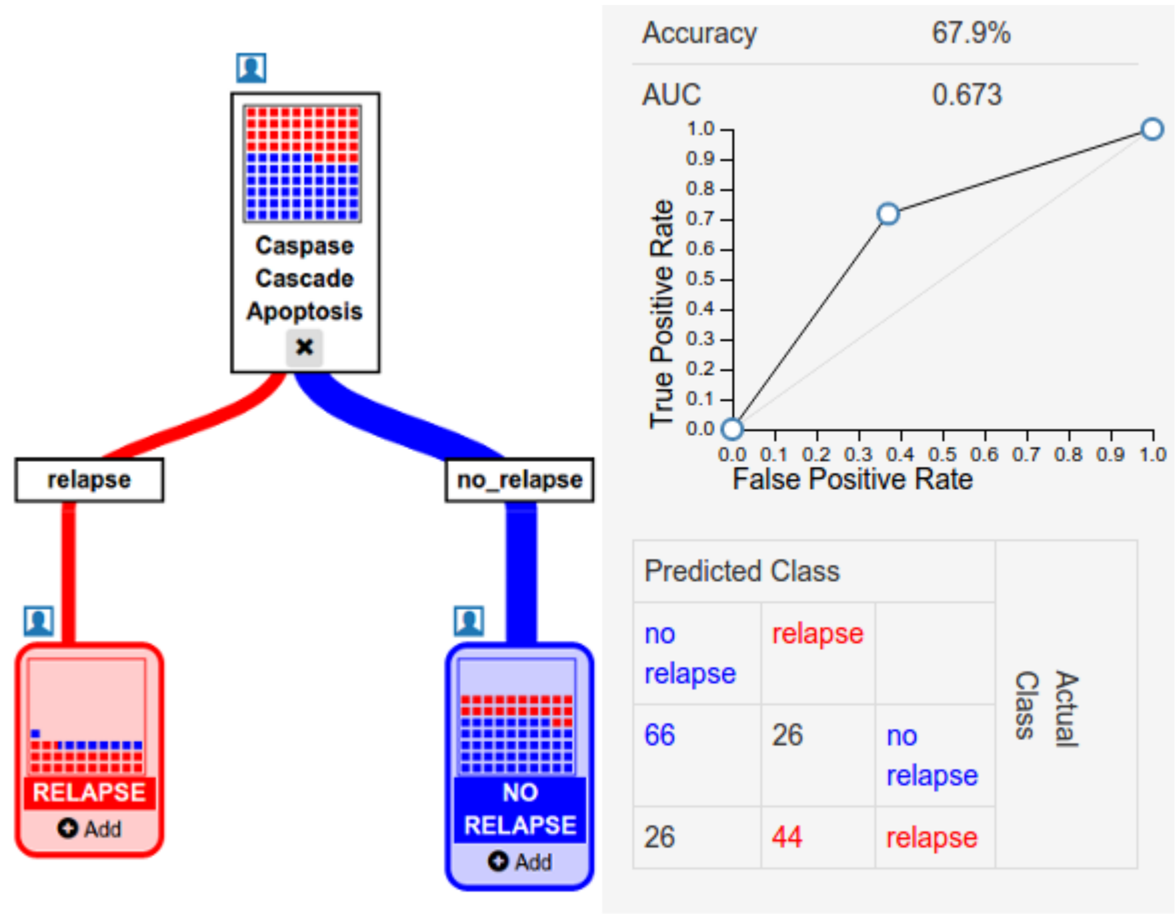

Figure 13: A SVM Classifier split node constructed using genes from the Caspase Cascade (triggered by Apoptosis) Pathway. Training Set: “Hormone-positive node-negative breast cancer survival prediction (Training set)”. Test Set: “Hormone-positive node-negative breast cancer survival prediction (Test set)”. The classifier has an accuracy of 67.9%.

References

Paik,S. et al. (2004) A multigene assay to predict recurrence of tamoxifen-treated, node-negative breast cancer. N. Engl. J. Med., 351, 2817–26.

Griffith,O. et al. (2013) A robust prognostic signature for hormone-positive node-negative breast cancer. Genome Med., 5:92.

Kamburov, A. et al. (2013) The ConsensusPathDB interaction database: 2013 update. Nucleic Acids Res. D793-D800.

Wilson, E. Probable Inference, the Law of Succession, and Statistical Inference DOI:10.1080/01621459.1927.10502953
